# The cardiometabolic "bottleneck" on cognitive function in aging

**DOI:** 10.1002/alz.70685

**Published:** 2025-09-08

**Authors:** Timothy Daly

**Affiliations:** ^1^ UMR 1219 Bordeaux Population Health Université de Bordeaux & INSERM Bordeaux France; ^2^ UMR 5164, ImmunoConcept University of Bordeaux & CNRS Bordeaux France; ^3^ Bioethics Program, FLACSO Argentina Buenos Aires Argentina

1

The analysis of Hayes and colleagues of postmortem neuropathology found that arteriolosclerosis—where the walls of the brain's small arteries (arterioles) thicken, harden, and reduce blood flow due to cardiometabolic conditions such as hypertension and diabetes—was an independent contributor to worsened cognition in people without dementia.^1^ Converging neuropathological and epidemiological evidence suggests that in aging individuals and populations, cardiometabolic risk factors represent an actionable choke point or bottleneck on cognitive function (Figure 1). As their study suggests, this is applicable to individuals without dementia but also across subtypes of dementia.

**FIGURE 1 alz70685-fig-0001:**
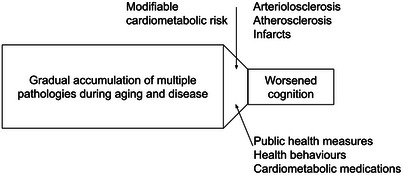
An individual and population perspective on cardiometabolic risk factors, which form a modifiable "bottleneck" on cognitive function across normal aging and different types of dementia due to cardiometabolic damage to brain blood vessels, suggesting the need for population and individual health measures to improve population brain health.

Although vascular dementia is the second most common cause of dementia as a nosological entity,^2^ most cases of later‐life cognitive decline are associated with the accumulation of multiple neurodegenerative and cerebrovascular pathologies.^3^ A recent population‐based Medicare analysis of over 20 million individuals found that 37% of dementia cases were associated with eight cardiometabolic risk factors (diabetes, hyperlipidemia, hypertension, stroke, chronic heart failure, ischemic heart disease, atrial fibrillation, and acute myocardial infarction).^4^

Moreover, a decades‐long cohort analysis and follow‐up with 12,274 participants found that 22%–44% of dementia risk by age 80 years was attributable to mid‐life and early late‐life vascular risk factors, with the risk higher in apolipoprotein E (*APOE*) ε4 non‐carriers.^4^ Even in small samples of focal cases of dementia, such as deterministic cases of familial Alzheimer's disease, World Health Organization (WHO)–defined high levels of physical activity have been associated with up to 15 years longer cognitive function.^5^ Taken together, these data suggest that cardiometabolic factors are responsible for life‐changing amounts of variability in cognitive decline, even in individuals with genetic profiles for earlier dementia onset.

It is important to note that cardiometabolic risk factors are modifiable, and epidemiological and neuropathological evidence suggests that indirect action against them may already be bearing significant public health gains. Indeed, it is now well known that in birth cohorts in high‐income countries from the 20th century, age‐specific dementia prevalence rates have been decreasing by over 10% per decade.^6^ These changes have not been associated with reduced neurodegenerative pathologies such as those associated with Alzheimer's disease, but rather “substantial decreases” in atherosclerosis and arteriosclerosis.^7^ This period coincides with reductions in health inequalities,^8^ suggesting that broad measures to improve access to education and improve cardiovascular health^3^ within the population have a population‐level impact on these vascular pathologies. Finally, there is now growing interest in cardioprotective antidiabetic drugs such as semaglutide and metformin, which may exert protective effects on dementia risk through cardiometabolic mechanisms, now under study through randomized clinical trials and observational[Fig alz70685-fig-0001] studies.^9^

In conclusion, cardiometabolic risk, such as hypertension and diabetes, may be responsible for significant proportions of modifiable risk for cognitive decline through mechanisms including arteriolosclerosis and other forms of vessel damage across diverse groups, such as people without dementia and those with high genetic risk for dementia, such as familial Alzheimer's disease and *APOE* ε4 homozygotes. Further research and policy to address the cardiometabolic bottleneck on cognitive function should explore public health measures to reduce health inequalities, improve population‐wide physical health in line with WHO recommendations for brain health,^10^ and enhance access to cardioprotective medicines in populations at higher cardiometabolic risk.

## CONFLICT OF INTERESTS STATMENT

Dr. Timothy Daly has no conflicts of interest to declare. Author disclosures are available in the supporting information.

## Supporting information



Supporting Information‐alz70685‐sup‐0001‐ICMJE.pdf
